# Adenocarcinoma of the Colon Disguised as Abdominal Wall Abscess: Case Report and Review of the Literature

**DOI:** 10.1155/2018/1974627

**Published:** 2018-01-24

**Authors:** L. Attar, N. Trabulsi, A. A. Maghrabi, M. Nassif

**Affiliations:** Department of Surgery, Faculty of Medicine, King Abdulaziz University, Jeddah, Saudi Arabia

## Abstract

**Introduction:**

Abdominal wall invasion by cancerous cells arising from the colon with an overlying secondary infection that presents as an abdominal wall abscess has been encountered previously, but such a symptom is rarely the first presentation of colon cancer. There are very few cases reported in the literature.

**Case Presentation:**

In this case report, we present a case of a 66-year-old male presenting with abdominal wall abscess that was refractory to treatment. The patient later was found to have an abdominal wall invasion by an underlying colonic carcinoma.

**Conclusion:**

The purpose of this review is to set forth the proper approach when encountering such cases and emphasize on the significance of keeping a high index of suspicion. We also highlight the need for utilizing proper diagnostic imaging modalities prior to invasive intervention.

## 1. Introduction

Carcinoma of the colon has the ability to mimic any abdominal disease with a wide spectrum of presentations. Direct invasion of transverse colon adenocarcinoma into the abdominal wall is rarely encountered. We hereby report a case of transverse colon tumor presented as an abscess infiltrating the abdominal wall.

Because of the infrequency of this condition, our purpose with this review will set forth the proper approach in such cases to spread the awareness and improve the outcome.

## 2. Case Presentation

Our patient is a 66-year-old Eritrean gentleman, who presented to our emergency department with severe epigastric pain and a history of a growing abdominal wall mass. On systematic review, he reported anorexia and weight loss, with no history of alteration in bowel habits. The patient had no significant past medical history apart from this presentation.

Ten days prior to his presentation to our institution, he underwent an incision and drainage procedure of an abdominal wall abscess at an outside institution. The patient was discharged with outpatient dressing protocol and oral antibiotics.

**Figure 1:**
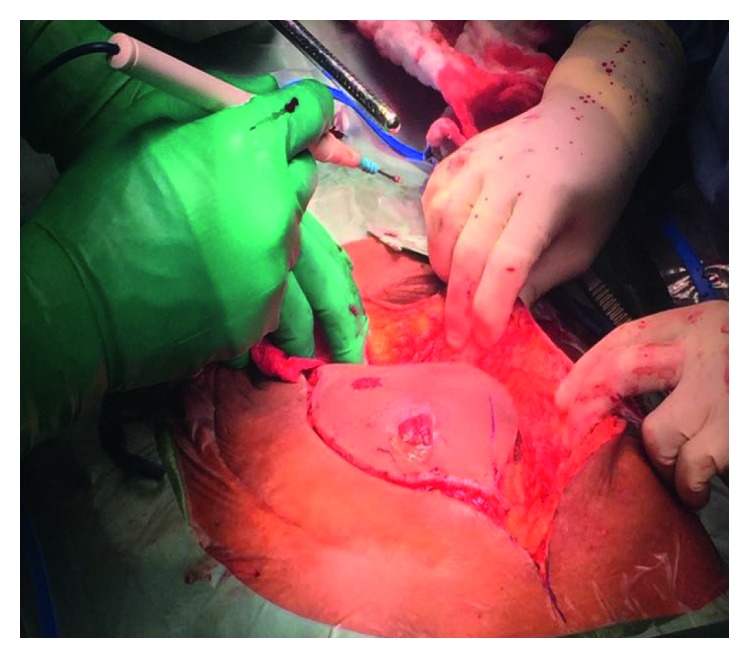
Patient on the table, presenting with abdominal wall abscess.

On examination, the patient was thin and cachectic, with a large tender warm swelling occupying the supraumbilical and epigastric regions. It measured about 10 × 15 cm in greatest dimension. There were two ulcerations on the surface of the swelling draining purulent discharge corresponding to the incisions done previously (Figure 1). There was no evidence of peritonitis or other significant physical findings.

Laboratory results revealed a hemoglobin level of 5.7 g/dl (normal range: 14–18), white blood count level of 13.3 K/*µ*L (normal range: 4.5–11.5), and carcinoembryonic antigen (CEA) level of 12.99 ng/ml (normal range: 0–3.4). Coagulation profile and liver function tests were within normal ranges. Wound culture showed mixed bacterial growth of *Escherichia coli* and *Klebsiella pneumoniae*.

## 2. Hospital Course

The patient was admitted for blood transfusion, intravenous hydration, and IV antibiotics while being worked up for the cause of this abdominal wall mass.

**Figure 2:**
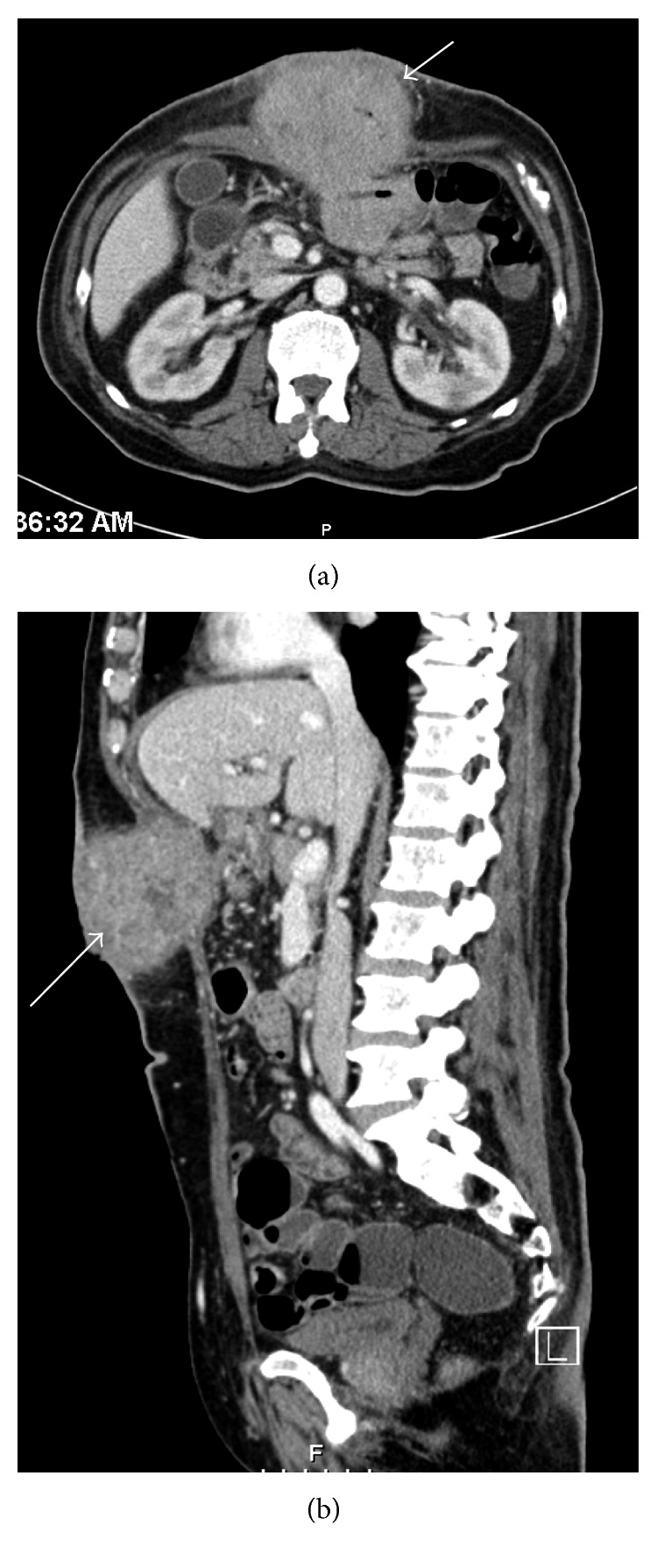
CT scan showing the mass, extending to the adjacent muscles and penetrating the abdominal wall.

Computed tomography of the abdomen and pelvis revealed a large soft tissue heterogeneous mass in the upper abdomen (measuring 9 × 10 × 10 cm). The mass was causing displacement of the stomach with no obstruction. It was inseparable from part of the small bowel, extending to the adjacent muscles and penetrating the abdominal wall (Figures 2(a) and 2(b)). However, the primary source could not be ascertained but was thought to be originating from the transverse colon. Thickening of the stomach wall was seen, and multiple paraaortic, retroaortic, mesenteric, and inguinal lymph nodes were noted. Computed tomography of the chest showed no evidence of thoracic disease.

Upper and lower gastrointestinal endoscopies were done. The upper scope was normal, and the lower scope was advanced up to the transverse colon but failed to advance beyond due to external compression causing complete luminal collapse.

Only gastric fold biopsy was taken, which revealed chronic superficial gastritis with no evidence of activity or malignancy.

**Figure 3:**
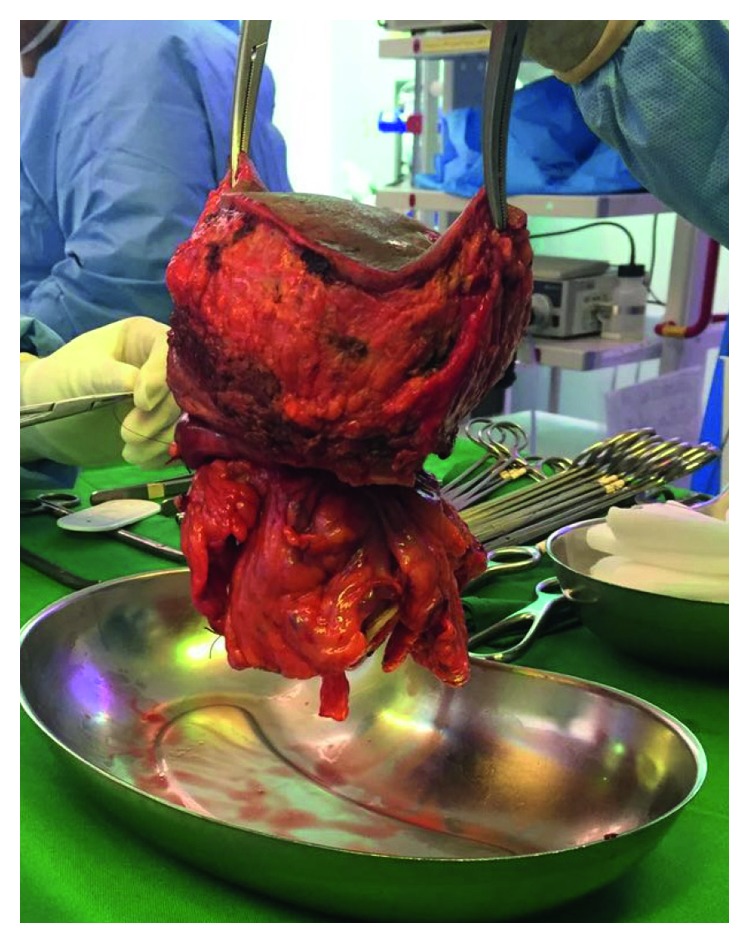
The surgical specimen after the excision (an extended right hemicolectomy, partial gastrectomy, and en bloc resection of the abdominal wall).

The patient underwent exploratory laparotomy through a midline incision in an elliptical fashion to include the affected abdominal wall part with the specimen; upon exploration, a malignant-looking mass was found to be adherent to the greater curvature of the stomach and the transverse colon. An extended right hemicolectomy with primary ileocolic anastomosis along with omentectomy, partial gastrectomy, and complete en bloc resection of the abdominal wall including the abscess area was performed (Figure 3). There was no evidence of liver metastasis or peritoneal dissemination.

The resulting defect in the fascia was large, 20 cm in its maximum dimension. The fascia was approximated after undermining. Abdominal wall reconstruction was successfully carried out by placing a double-layer synthetic mesh (Vicryl) in a tension-free manner, and the skin was closed over it. The patient had an uneventful recovery.

Postoperative histopathological examination revealed a transverse colon moderately differentiated adenocarcinoma with direct invasion to the abdominal wall and stomach. All surgical margins were free of disease, and fifteen lymph nodes were retrieved and were found to be negative for malignancy. The pathological staging was (T4b, N0, M0). The final stage was Stage IIC according to American Joint Committee on Cancer (AJCC) staging. Follow-up CT scan was done on day 5, which showed subcutaneous midline anterior abdominal wall fluid collection with multiple air pockets with a tiny contrast focus noted at the inferior part representing enterocutaneous fistula. The follow-up CEA level was 3.45 ng/ml. The patient was scheduled for chemotherapy cycles with the oncologist. The patient received 6 cycles of chemotherapy (capecitabine 1500 mg tablet BID and Xelox). He is being followed up and free of recurrence after almost 19 months.

## 3. Discussion

Locally advanced colorectal cancers, a subgroup of colorectal tumors that invade adjacent organs without distant metastases, account for 5%–22% of all colorectal cancers [1]. Locally advanced colon cancer with direct invasion into adjacent organs or that spreads along the tissue planes may result in formation of abscesses in unusual locations such as the abdominal wall, and sometimes, this can be the first presentation [2–4]. The abdominal wall is more likely to be invaded by tumors arising from the intraperitoneal part of the colon. Although the majority of these complications rarely occur, familiarity with the various manifestations of colon cancer complications will facilitate making an accurate diagnosis and administering prompt management in these situations. Abscess formation arising from colon cancer is a rare complication that occurs in 0.3–4% of the cases [2–4].

When encountering such a case, early diagnosis, appropriate drainage, and definitive management may reduce patient morbidity and mortality rates especially if the patient was in poor condition in which elimination of the source of sepsis may be lifesaving [5]. Confirming a diagnosis of an underlying colon cancer preoperatively poses a surgical challenge. Inaccurate diagnosis without the recognition of the underlying malignancy may lead to incomplete treatment. The diagnosis may be definitively established intraoperatively, and some cases underwent preoperative drainage without confirming the diagnosis [3]. Multivisceral resection is planned before surgery in only a minority of patients; the decision to perform a multivisceral resection for primary colorectal carcinoma is usually made during surgery in most instances.

Patients presented with atypical abdominal wall abscess should be further evaluated by imaging modalities. Abdominal computed tomography (CT) is a very valuable tool and the key in making the diagnosis and planning for surgery. Computed tomographic colonography (CTC) provides comprehensive examination for preoperative evaluation of patients with suspicious lesions or with colorectal cancer. Sali et al. [6] described CTC as a “one-stop-shop” examination for preoperative assessment; it is accurate in detection and localization of the tumor with the chance to evaluate the entire colon and permits staging of extracolonic tumor spread, both locoregional and distant. Its role, technique, and limitations are described well in his study. Kim et al. [7] reviewed the CT appearance of the colonic complications (including the abscess) associated with colon cancer and specified the important factors and morphological appearance that help in distinguishing the malignancy from the inflammatory or benign abscesses. A multitude of studies have described CTC as feasible, safe, and well tolerated.

En bloc resection is the treatment of choice in the majority of such cases, given the fact that R0 resection (complete resection, margins histologically negative, and no residual tumor left after resection) [8, 9] with free surgical margin does not adversely affect the prognosis. Exception will be made in few cases in which complete en bloc resection cannot be performed; in such circumstances, resection of the primary tumor with en bloc partial resection of the adherent parietal wall should be performed if possible [10]. Complete radical resection should be achieved as possible in good surgical candidates. The procedure has a low morbidity and mortality even in the elderly [11–15].

### 3.1. Prognostic Measures and Survival Rate in Patients with Locally Advanced Colon Cancer

Little is known about the outcome of multivisceral resection for colorectal cancer. Complete, microscopic negative margin resection is a critical predictor factor for long-term survival. In a prospective study conducted by Lehnert et al., 201 patients (139 colon cancer and 62 rectal cancer) underwent en bloc resection for locally advanced tumors [16]. After multivariate analysis, they demonstrated that operative blood loss, age, and stage were independent prognostic factors. Histologic tumor infiltration, the number of resected organs, or surgical experience did not influence chances of survival. No patient with incomplete resection (R1, R2) survived for five years. Multiple studies [11, 16, 17] supported that the long-term outcome after multivisceral resection is comparable and similar to that after the standard operation. The 5-year survival rate for curative resection was found to be 76.6% versus 79.5% for standard resection with higher morbidity rates among patients who underwent multivisceral resection [17]. Other studies have confirmed that reduction in the incidence of blood transfusion may contribute to better patients' survival, and poorer prognosis was associated with advanced cancer stage, the number of metastatic lymph nodes, and perforation proximal to the cancer site [17, 18]. Locally advanced colon tumors with perforation at the tumor site usually tend to require emergency surgery with a higher 30-day mortality rate [19]. The mortality rate after emergency operation was found to be 23% [20]. Despite this, better outcome could be reached by adequate management of the initial emergency situation (e.g., sepsis).

The role of chemotherapy and radiation holds promise in facilitating curative resection and improving postresection survival in locally advanced colorectal cancer.

### 3.2. Role of Prophylactic Hyperthermic Intraperitoneal Chemotherapy (HIPEC)

The percentage of developing peritoneal metastasis (PM) after complete resection of colorectal cancer is 10% of all patients [21]. Patients with PM used to have very poor outcomes with very low 5-year survival rates [22–25]. The emerging role of the multimodal strategy of cytoreductive surgery (CRS) and hyperthermic intraperitoneal chemotherapy (HIPEC) and systemic chemotherapy improves the overall survival and is considered as the standard treatment for colorectal peritoneal metastasis in selected patients [21, 26]. This multimodal approach prolonged the survival up to 64 months (6 months without treatment) with a mean 5-year survival of 51% with complete resection and a disease-free survival at 5 years of 16% [26]. The 5-year survival with use of systemic chemotherapy alone was found to be 20% [23]. Verwaal et al. [23] showed in a randomized controlled trial that patients undergoing CRS and HIPEC have better survival especially in those who had complete cytoreduction without any residual disease with a 5-year survival of 45%. When modern chemotherapeutic agents were compared to CRS and HIPEC, patients in the experimental group showed a 5-year survival of 51% [25]. If early detection achieved, PM patients with low peritoneal carcinomatosis index (PCI) (≤5) can be treated properly (by CRS + HIPEC) with a markedly high survival rate and even could have better prognosis than those with liver metastasis with a 3-year survival of 89% [27]. Huang et al. [28] supported these data by reviewing 60 low PCI PM patients and observed a 5-year survival rate of 54.7% with no mortality. Sugarbaker established survivorship using the PCI. Five-year survival was 50% in colon cancer patients with carcinomatosis with a PCI less than 10, 20% for 11–20, and 0% in those with a PCI score greater than 20 [29]. For this reason, all efforts should be made to detect patients with PM at the earliest stage. Unfortunately, the current diagnostic workup tools lack the ability to detect PM at very early stage. If detection occurred, those patients usually present with high PCI (>20) [30], and CRS and HIPEC strategy offers a poor outcome [31]. Proposing a systematic second-look surgery is the only way to achieve early detection, and this is considered as an invasive approach and cannot be proposed to all patients but only to high-risk selected populations. Honoré et al. [21] defined the characteristics of patients with primary colorectal cancer identified in the literature as being at high risk of developing peritoneal metastasis: visible evidence of peritoneal metastasis (70%), ovarian metastasis (60%), perforated cancer (spontaneous or iatrogenic perforation) (50%) [21]. The estimated incidence of peritoneal metastasis as a recurrence had been observed in a study conducted by Sugarbaker [32]. This will make a conclusion that anticipating the risk of relapse and providing a second-look surgery for patients considered at high risk to develop PM could significantly change the survival. That was supported by the notable study conducted by Elias et al. [33]. Their aim was to assess the role of second-look surgery and HIPEC one year after curative resection in asymptomatic high-risk patients based on the criteria mentioned in the previous study. PM was found and treated with complete surgery plus HIPEC in 23 of the 41 (56%) patients. The 5-year overall survival rate was 90%, and the 5-year disease-free survival rate was 44%.

In the light of these promising results, phase III studies were initiated to highlight the role of second-look surgery and administration of prophylactic HIPEC. A multicentric randomized controlled trial being run by Elias and his colleagues (ProphyloCHIP) [34] is comparing the simple follow-up to exploratory laparotomy plus HIPEC among 150 patients considered at very high risk of peritoneal relapse after a year of first intervention, both groups receiving systemic adjuvant therapy; the study will be completed in June 2019. Another Dutch trial COLOPEC [35] is assessing the effectiveness of adjuvant HIPEC in preventing the development of PM in patients with colon cancer at high risk of peritoneal recurrence. In this trial, the patients are randomized into two groups: adjuvant HIPEC followed by routine adjuvant systemic chemotherapy in the experimental arm versus systemic chemotherapy only in the control arm.

Locally advanced colorectal cancer may require an intraoperative decision for en bloc resection of surrounding organs or structures to achieve complete tumor removal. Long-term survival after surgery becomes achievable with all efforts being made to reach complete resection. The prevention of a disease process has always been superior to the treatment of the same disease; early PM detection and treatment at an earlier stage prolong the survival.

In summary, locally advanced colon cancer still poses challenges when it comes to achieving the best therapeutic approach. In those presenting with perforation, a high index of suspicion early on helps early identification of underlying malignancy in otherwise previously not known patients. A multimodal approach helps optimize the patient's treatment and ultimately survival.

## Data Access

All data and material presented in the article are available upon request.
